# Elevated red blood cell distribution width levels predict depression after intracerebral hemorrhage: A 3-month follow-up study

**DOI:** 10.3389/fneur.2023.1077518

**Published:** 2023-04-04

**Authors:** Xianping Zhou, Yaqiang Li, Zhongbo Sun, Li Mu, Yaoyao Ma

**Affiliations:** ^1^Department of Laboratory, Bozhou Hospital Affiliated to Anhui Medical University, Bozhou, China; ^2^Department of Neurology, People's Hospital of Lixin County, Bozhou, China; ^3^Department of Neurosurgery, First Affiliated Hospital of Anhui University of Science and Technology (First People's Hospital of Huainan), Huainan, China

**Keywords:** inflammation, red blood cell distribution width, depression, stroke, intracerebral hemorrhage

## Abstract

**Objectives:**

Inflammation vitally impacts the progression of depression resulting from intracerebral hemorrhage (ICH), while red blood cell distribution width (RDW) marks inflammatory-related diseases. The present study aimed at evaluating how RDW affects depression after ICH.

**Methods:**

From prospective analyses of patients admitted to our department between January 2017 and September 2022, ICH patients with complete medical records were evaluated. The 17-item Hamilton Depression (HAMD-17) scale was used for measuring the depressive symptoms at 3 months after ICH. Diagnosis of post-ICH depression was conducted for patients based on the Diagnostic and Statistical Manual of Mental Disorders, Fifth Edition (DSM-V) criteria.

**Results:**

A total of 438 patients were enrolled in the study, out of which 93 (21.23%) patients had PSD at the 3-month follow-up. Accordingly, patients with depression had higher RDW levels (13.70 [IQR: 13.56–13.89] vs.13.45 [IQR: 12.64–13.75], *p* < 0.001) at admission compared with those without depression. In multivariate analyses, RDW was used for independently predicting the depression after ICH at 3 months (OR: 2.832 [95% CI: 1.748–4.587], *p* < 0.001). After adjusting the underlying confounding factors, the odds ratio (OR) of depression after ICH was 4.225 (95% CI: 1.686–10.586, *p* = 0.002) for the highest tertile of RDW relative to the lowest tertile. With an AUC of 0.703 (95% CI: 0.649–0.757), RDW demonstrated a significantly better discriminatory ability relative to CRP and WBC. RDW as an indicator for predicting depression after ICH had an optimal cutoff value of 13.68, and the sensitivity and specificity were 63.4% and 64.6%, respectively.

**Conclusions:**

Elevated RDW level predicted post-ICH depression at 3 months, confirming RDW as an effective inflammatory marker for predicting depression after ICH.

## 1. Introduction

Intracerebral hemorrhage (ICH) is an extremely dangerous type of stroke, which takes up nearly 10%−30% of all illnesses and deaths related to stroke (1). The prevalence of stroke in most provinces in China has increased from 2.28% to 2.52% in the past 7 years (2013–2019) (2). Poststroke depression (PSD) is one of the most frequent psychosomatic disorders after stroke (such as ICH) and can lead to lower quality of life, lower cognitive function, weak functional outcomes, and elevated mortality (3–5). Accumulating studies have revealed that approximately 20% of ICH survivors presented signs of depression (6, 7). Therefore, it is still necessary to further explore factors that predict the early diagnosis of PSD as well as the potential pathophysiological mechanisms of PSD (8).

Currently, PSD is associated with biological factors from many perspectives, namely, the inflammatory response, oxidative stress, nitrosative stress pathways, neurotransmitter systems, neurotrophin, and neurogenesis regulation, as well as hypothalamic–pituitary–adrenal axis modulation (9, 10). According to extensive reports, high levels of pro-inflammatory and inflammatory markers impacted the inflammatory response to depression, namely, interleukin-1(IL-1), IL-18, IL-6, tumor necrosis factor-α (TNF-α), IFN-γ, and C-reactive protein (CRP) (11–13). Consequently, animal experiments have also confirmed the association of inflammation with the development of PSD (14). In addition, increased systemic immune-inflammation index (SII), neutrophil-to-lymphocyte ratio (NLR), platelet-to-lymphocyte ratio (PLR), and derived neutrophil-to-lymphocyte ratio (dNLR), especially the SII at admission, were considerably related to PSD and may assist in the early detection of PSD from a prognostic aspect (15). Inflammation has also been indicated recently to facilitate the depression development after ICH by the toll-like receptors (TLRs) signal pathway, nuclear factor kappa-B (NF-kB) mediated signal pathway, peroxisome proliferator-activated receptor (PPAR)-γ-dependent signal pathway, etc. (16).

Red blood cell distribution width (RDW) constitutes the complete blood count as well as serves as an accessible marker of disease related to inflammation. It represents the coefficient of variation of circulating erythrocyte size or erythrocyte volume distribution and may represent the states of chronic systemic inflammation, malnutrition, and microcirculatory disorders (17). Numerous clinical studies have investigated how RDW affects the prognosis of some vascular diseases, namely, in ICH, hemorrhagic transformation for patients with acute ischemic stroke who had received intravenous thrombolysis therapy, acute non-traumatic subarachnoid hemorrhage, acute myocardial infarction, symptomatic chronic heart failure, and ischemic stroke (18–22).

Post-ICH depression presented a relationship with the worsening disability in the late stages, which was irrelevant to the initial bleeding severity (7). Additionally, higher-level RDW could independently predict the occurrence of depression after acute cerebral ischemia (23, 24) and predicted the risk of coronary artery disease for patients with major depression disorder (25). Elevated RDW is correlated with the occurrence of stroke and strongly predicts both cardiovascular and all-cause mortalities in patients with known strokes (26). A literature review has also revealed that the higher RDW independently predicted poor outcomes in patients with ischemic stroke (IS), carotid atherosclerosis, or cerebral embolism (27). In addition, a comprehensive meta-analysis of 31 studies revealed that higher RDW was associated with unfavorable functional outcomes, either at discharge or at 90 days (28). To date, there have been no studies exploring the effect of RDW on post-ICH depression. Therefore, the study was aimed at investigating the relationship between RDW and PSD, as well as further exploring how RDW predicted depression at 3 months after ICH.

## 2. Materials and methods

### 2.1. Subjects

**Figure 1:**
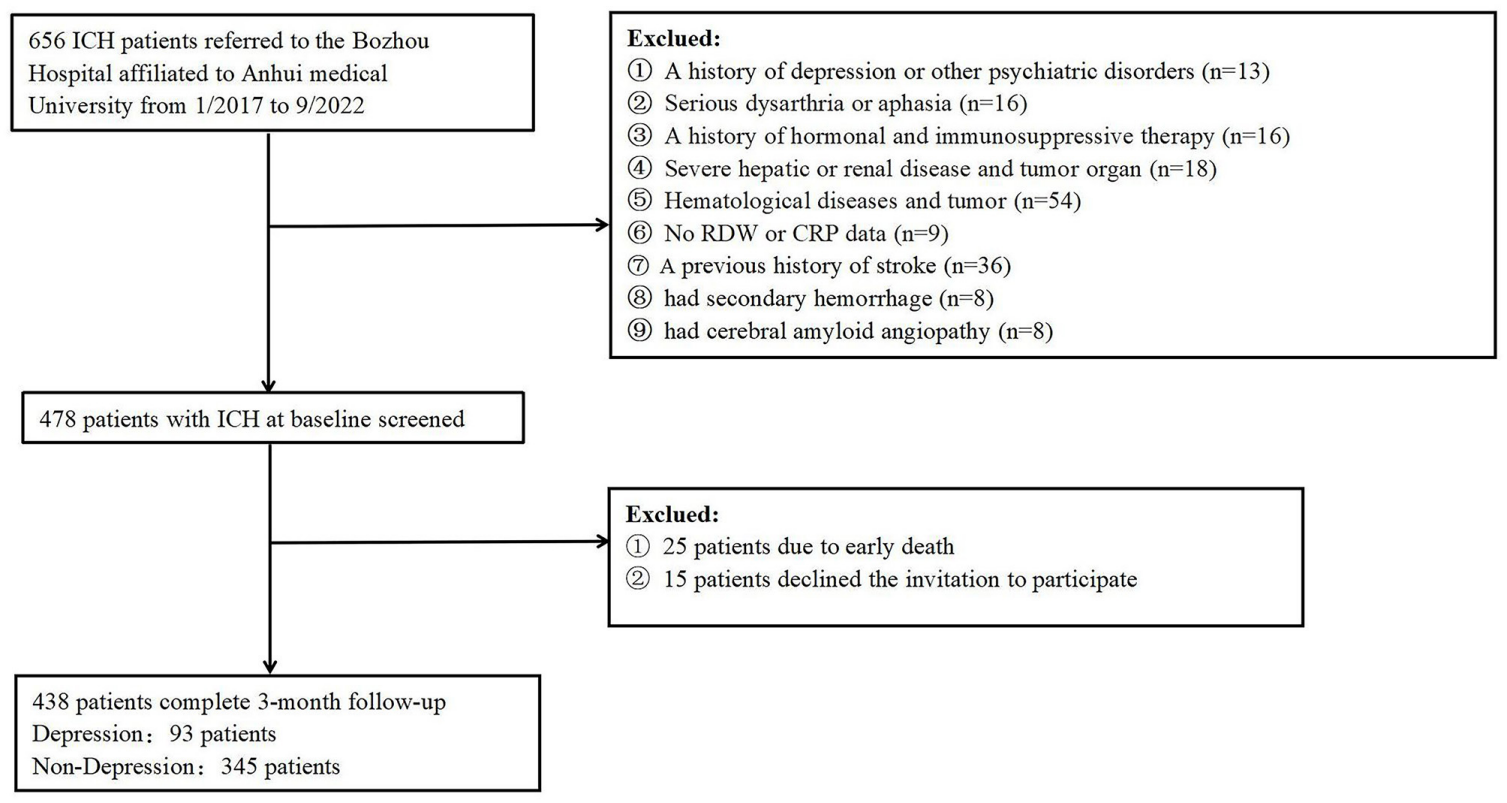
Flowchart of participant selection.

From January 2017 to September 2022, patients with ICH were consecutively enrolled from the Bozhou Hospital, Anhui Medical University, Bozhou, China in this prospective observational study. Within 24 h after all suspected ICH patients were admitted to the hospital, computed tomography (CT) confirmed the diagnosis. Patients were not included if they (1) had a hemorrhagic transformation of cerebral infarction, primary intraventricular hemorrhage, hemorrhage due to arteriovenous malformation, hemorrhage due to aneurysm, and secondary hemorrhage due to anticoagulant therapy; (2) had no CRP or RDW data; (3) had hematological diseases and renal or liver diseases; (4) had hormonal and immunosuppressive therapy history with 3 months prior to ICH onset; (5) concurrently had a disease that was possibly affecting the RDW value, such as active infection, chronic inflammatory disease, malignancy, and autoimmunological diseases; (6) once suffered major depression disorder (MDD) or other psychiatric disorders; (7) once suffered severe aphasia, dysarthria, and cognitive impairment that made it impossible to complete the psychological scale assessment; (8) had a previous history of stroke; (9) had cerebral amyloid angiopathy; or (10) had undergone surgical treatment. At last, the study included 438 cases with ICH in total (Figure 1). This study has obtained the approval of the institutional ethics review board of the participating center and also all participants' written informed consent following the Helsinki Declaration of 1975.

### 2.2. Data collection

We collected the sociodemographic and clinical characteristics regarding ICH patients on admission, such as age, sex, marital status, educational level, vascular risk factors (hypertension, diabetes mellitus, coronary heart disease, atrial fibrillation, current smoking habit, alcohol consumption, and prior stroke), hematoma volume and location, and adopted a standard case report form. The National Institutes of Health Stroke Scale (NIHSS) was used for assessing the stroke severity on the first day (time 1) and on day 90 (time 2) after the admission. Impairment of consciousness was categorized by the use of the Glasgow Coma Scale (GCS) score. The Barthel index (BI), the modified Rankin Scale (mRS), and the Mini-Mental State Exam (MMSE) were used for assessing the functional outcome and the cognitive functions after 90-day follow-up, respectively (29–31). ICH volume was measured by ABC/2 technology (A = greatest hemorrhage diameter by CT, B = diameter perpendicular to A, and C = the approximate number of CT slices with hemorrhage multiplied by the slice thickness).

### 2.3. Blood collection and laboratory test

The study enrolled participants with ICH within 24 h of onset. We collected patients' blood samples after an 8-h fast in the morning at room temperature. Sysmex XE-2100 Hematology Automated Analyzer was used for measuring the RDW, white blood cell (WBC), mean corpuscular volume (MCV), mean corpuscular hemoglobin (MCH), mean corpuscular hemoglobin concentration (MCHC), platelet (PLT), hemoglobin (Hb), and red blood cell (RBC) at our hospital's laboratory. The serum CRP, glucose (G), total cholesterol (TC), triglycerides (TG), high-density lipoprotein cholesterol (HDL-C), low-density lipoprotein cholesterol (LDL-C), homocysteine (Hcy), apolipoprotein A (ApoA), and apolipoprotein B (ApoB) were measured by AU5811 automatic biochemical analyzer (Beckman Coulter, USA) in the clinical laboratories of our hospital. All methods and procedures followed in this study strictly conformed to relevant instructions. To minimize assay variation, professional clinical technicians who did not know the clinical outcomes or neuroimaging findings took charge of randomly analyzing all samples in duplicate on the same day.

### 2.4. Psychological measurement

The primary endpoint referred to the psychological evaluation 3 months after the admission, which was carried out at 3 months by two trained neurological physicians who did not know the laboratory results of patients. All participants who were diagnosed with major, moderate, or minor depression received the structured clinical interview following the Diagnostic and Statistical Manual of Mental Disorders, 5th edition (DSM-V) (SCID-I-R). We investigated the inter-rater reliability for 200 patients and confirmed the kappa as 0.90 (YQ Li and ZB Sun). The HAMD-17 scale was used for measuring depressive severity and a HAMD-17 scale score > 7 was reported for PSD (32).

### 2.5. Statistical analysis

SPSS 23.0 statistical software (SPSS, Inc., Chicago, IL, USA) was used for the statistical analyses. Data for continuous variables were in the form of mean ± standard deviation (SD) or median (interquartile range), which was decided by whether the tested data presented normal or non-normal distribution. The Chi-square test was used in evaluating categorical variables. In the case of conducting an abnormal distribution test on the continuous variables, the Kruskal–Wallis test and the Mann–Whitney U test were used for comparing the difference between the three groups and that between two groups, respectively. Student's *t*-test or one-way analysis of variance (ANOVA) were used for analyzing the normally distributed continuous variables. We included the variables with a *p*-value < 0.5 confirmed by the univariate analysis in the final multivariable analysis. Multivariate logistic regression analysis was used for determining the risk factors independently for predicting depression 3 months after ICH. Bivariate correlations were analyzed with Pearson correlation coefficient test or Spearman rank correlation test analyses. The admission RDW was taken into account for dividing patients into tertiles (T1: ≤ 12.95, T2: 12.97–13.69, and T3: ≥13.7). We applied three models for the multivariable regression analyses, for recognizing the factors that could predict post-ICH depression, with model 1 targeting age, sex, education years, and marital status; model 2 targeting model 1 as well as vascular risk factors; model 3 targeting variables with a *p* < 0.05 confirmed by the univariate analyses (WBC, CRP, BGLs, baseline NIHSS score, mRS score, and BI score). In addition, the association was in the form of OR with 95% CI. Besides, a receiver operating characteristic curve (ROC) analysis assisted in identifying the cutoff point on the RDW levels on admission, which could be the most sensitive and specifically serve for predicting the post-ICH depression at the 3-month follow-up. We calculated the AUC regarding RDW for measuring the test accuracy; *p* < 0.05 was reported to be statistically significant.

## 3. Results

### 3.1. Baseline characteristics exhibited by patients in depression and non-depression groups

The study included ICH patients presented at the participating center from January 2017 to September 2022. We first enrolled 656 participants, followed by excluding 218 participants, including 15 participants who were not followed up at 3 months, 25 patients who died, 9 participants who did not provide their RDW or CRP data, and 169 participants who conformed to the exclusion criteria, such as a history of depression or other psychiatric disorders, a history of hormonal and immunosuppressive therapy, serious dysarthria or aphasia, hepatic or renal disease, hematological diseases, tumor, as well as secondary hemorrhage, etc. Lastly, the study yielded 438 patients (172 women, aged 66.16 ± 8.10 years), of which 93 (21.23%) patients were included in the depression group after ICH and 345 (78.77%) patients were included in the non-depression group after ICH. Relative to the patients in the depression group after ICH, those in the non-depression group presented higher baseline NIHISS1 scores (*p* < 0.001), higher NIHISS2 scores (*p* < 0.001), higher WBC (*p* < 0.001), higher CRP (*p* < 0.001), higher RDW (*p* < 0.001), higher mRS scores (*p* < 0.001), and lower BI scores (*p* < 0.001). Among hematoma locations, the basal ganglia (*p* = 0.014) was considerably related to the depression risk. Table 1 compares the baseline characteristics between the two groups of patients.

**Table 1:** Clinical and demographic characteristics of patients in the depression and non-depression groups.

**Variables**	**Total (*n* = 438)**	**Depression after ICH**		***p-*value**
		**Depression (*****n*** = **93)**	**Non-depression (*****n*** = **345)**	
**Demographic characteristics**				
Sex, female, *n* (%)	172 (39.27)	44 (47.31)	128 (37.10)	0.074
Age, years, mean ± SD	66.16 ± 8.10	66.30 ± 7.96	65.65 ± 8.62	0.491
Education years, median (IQR)	5 (3–8)	5 (3–8)	5 (3–8)	0.255
Marital status, married, n (%)	416 (94.98)	88 (94.62)	328 (95.07)	0.860
**Vascular risk factors (%)**				
Hypertension	281 (64.16)	55 (59.14)	226 (65.51)	0.256
Diabetes mellitus	132 (30.14)	31 (33.33)	101 (29.28)	0.449
Coronary heart disease	52 (11.87)	14 (15.05)	38 (11.01)	0.285
Atrial fibrillation	38 (8.68)	12 (12.90)	26 (7.54)	0.150
current smoking	133 (30.37)	30 (32.26)	103 (29.86)	0.655
Alcohol consumption	155 (34.70)	33 (35.48)	119 (34.49)	0.859
Hematoma volume, ml, median (IQR)	7 (4–10)	8 (5–9)	6 (3–10)	0.092
**Laboratory findings (IQR)**				
WBC, × 10^9^/L, median (IQR)	6.48 (5.62–7.75)	7.53(6.18–8.75)	6.29 (5.39–7.44)	^***^
CRP, mg/L, median (IQR)	6.11 (5.26–7.48)	7.22 (6.04–9.20)	5.96 (5.17–7.00)	^***^
Plt, × 10^9^/L, mean ± SD	213 ± 58	203 ± 59	216 ± 57	0.055
MCV, fl, mean ± SD	86.95 ± 5.97	87.56 ± 6.02	86.78 ± 5.96	0.281
MCH, pg, median (IQR)	29.58 (27.00–32.20)	29.50 (26.75–33.10)	29.60 (27.00–32.00)	0.467
MCHC, g/L, mean ± SD	328.79 ± 31.12	328.61 ± 14.12	328.83 ± 34.31	0.952
RBC, × 10^12^/L, mean ± SD	4.81 ± 0.54	4.86 ± 0.49	4.80 ± 0.55	0.345
Hb, g/L, mean ± SD	139 ± 11	139 ± 11	139 ± 10	0.996
RDW, %, median (IQR)	13.56 (12.72–13.78)	13.70 (13.56–13.89)	13.45 (12.64–13.75)	^***^
Glucose, mmol/L, median (IQR)	5.30 (4.70–6.80)	5.60 (4.70–6.75)	5.2 (4.70–6.80)	0.329
TG, mmol/L, mean ± SD	1.67 ± 1.27	1.62 ± 1.51	1.68 ± 1.20	0.962
TC, mmol/L, mean ± SD	4.64 ± 1.49	4.59 ± 1.06	4.65 ± 1.59	0.916
HDL-C, mmol/L, mean ± SD	1.09 ± 0.52	1.18 ± 0.96	1.06 ± 0.31	0.241
LDL-C, mmol/L, mean ± SD	2.57 ± 0.87	2.64 ± 0.84	2.55 ± 0.88	0.422
ApoA, g/L, mean ± SD	1.28 ± 0.27	1.30 ± 0.24	1.28 ± 0.28	0.666
ApoB, g/L, mean ± SD	0.90 ± 0.39	0.92 ± 0.27	0.89 ± 0.41	0.546
Hcy, μmol/L, median (IQR)	12.76 (10.29–16.30)	13.01 (10.81–16.46)	12.76 (10.19–16.39)	0.415
**Hematoma location**, ***n*** **(%)**				
Frontal lobe	66 (15.07)	10 (10.75)	56 (17.23)	0.190
Parietal lobe	52 (11.87)	10 (10.75)	42 (12.17)	0.707
Temporal lobe	129 (29.45)	26 (27.96)	103 (29.86)	0.722
Occipital lobe	62 (14.16)	11 (11.83)	51 (14.78)	0.468
Basal ganglia	174 (39.73)	47 (50.53)	127 (36.81)	0.016
Brainstem	31 (7.08)	8 (8.60)	20 (6.67)	0.182
Cerebellum	52 (9.59)	7 (7.53)	45 (13.04)	0.144
Concurrent ventricular hemorrhage	31 (7.08)	10 (10.75)	21 (6.09)	0.142
**Neuropsychological function**				
NIHSS1 score, median (IQR)	6 (4-9)	10 (8-13)	5 (4-8)	^***^
NIHSS2 score, median (IQR)	3 (1–5)	6 (4–8)	2 (1–4)	^***^
MMSE score, median (IQR)	22 (18–26)	20 (17–26)	22 (18–26)	0.788
GCS score, median (IQR)	15 (15–15)	14 (15–15)	15 (15–15)	0.124
BI score, median (IQR)	65 (45–85)	50 (40–65)	70 (50–90)	^***^
mRS score, median (IQR)	2 (2–3)	4 (3–4)	2 (2–3)	^***^
HAMD score, median (IQR)	5 (2–8)	7 (3–12)	4 (2–6)	^***^

^***^p < 0.001.

### 3.2. Baseline characteristics exhibited by all patients in RDW tertiles

Tertiles of the RDW level were taken into account for dividing all patients into three subgroups, ensuring that each subgroup had sufficient patient categories from 10.96 to 16.30 (T1, 146 patients; T2, 146 patients; and T3, 146 patients). The cutoff values (COV) for stratifying the RDW into tertiles were T1, 10.96–12.95; T2, 12.97–13.69; and T3, 13.70–16.30. Ascending tertiles of RDW reported lower BI scores (*p* = 0.001), higher RDW (*p* < 0.001), higher NIHSS1 scores (*p* < 0.001), higher NIHISS2 scores (*p* < 0.001), higher HAMD scores (*p* = 0.024), higher CRP (*p* < 0.001), higher hematoma volume (*p* < 0.001), and higher WBC (*p* = 0.007) (Table 2). The depression and non-depression groups presented significant differences in terms of the RDW (χ^2^ = 25.88, *p* < 0.001). For the depression group, the percentage of patients in the lowest tertile (10.96–12.95) and the highest tertile (13.70–16.30) were remarkably lower and higher, respectively. Besides, there were 11 (11.83%), 37 (39.78%), and 45 (48.39%) patients with depression after ICH in tertile 1, tertile 2, and tertile 3, respectively (Table 3).

**Table 2:** Baseline characteristics exhibited by Patients With ICH according to RDW tertiles.

**Variables**	**RDW**			***p* value**
	**T1 (** ≤ **12.95**, ***n*** = **146)**	**T2 (12.97-13.69**, ***n*** = **146)**	**T3 (**≥**13.7**, ***n*** = **146)**	
**Demographic characteristics**				
Sex, female, *n* (%)	48 (32.88)	66 (45.21)	88 (60.27)	0.097
Age, years, mean±SD	68 (62–71)	68 (63–72)	66 (61–70)	0.103
Education years, median (IQR)	5 (3–8)	5 (3–7)	5 (3–8)	0.587
Marital status, married, *n* (%)	140 (95.89)	139 (95.21)	137 (98.84)	0.715
**Vascular risk factors (%)**				
Hypertension	101 (69.18)	88 (60.27)	92 (63.01)	0.267
Diabetes mellitus	38 (26.03)	47 (32.19)	47 (32.19)	0.415
Coronary heart disease	17 (11.64)	17 (11.64)	18 (12.33)	0.658
Atrial fibrillation	8 (5.48)	9 (6.16)	15 (10.27)	0.235
current smoking	47 (32.19)	40 (27.40)	46 (31.51)	0.629
Alcohol consumption	52 (35.62)	52 (35.62)	48 (32.88)	0.851
Hematoma volume, ml, median (IQR)	5 (2–8)	4 (7–11)	8 (4–11)	^***^
**Laboratory findings (IQR)**				
WBC, × 10^9^/L, median (IQR)	6.10 (5.26–7.44)	6.66 (5.73–7.92)	6.79 (5.74–8.15)	0.007
CRP, median (IQR)	5.62 (4.68–6.55)	6.14 (5.43–7.61)	6.77 (5.95–8.37)	^***^
Plt, × 10^9^/L, median (IQR)	212 (174–250)	219 (182–255)	213 (173–241)	0.308
MCV, fl, mean ± SD	86.58 ± 5.68	87.34 ± 5.81	86.93 ± 6.42	0.555
MCH, pg, median (IQR)	29 (27–32)	29.89 (27.40–33.23)	29.65 (26.90–31.43)	0.852
MCHC, g/L, median (IQR)	330.70 (320.00–339.65)	331.60 (323.75–342.00)	328.30 (314.80–341.60)	0.119
RBC, × 10^12^/L, median (IQR)	4.94 (4.54–5.26)	4.78 (4.51–5.23)	4.54 (3.79–5.36)	0.207
Hb, g/L, median (IQR)	141 (134–145)	140 (134–145)	138 (132–144)	0.268
RDW, %, median (IQR)	12.57 (11.75–12.80)	13.56 (13.24–13.68)	13.87 (13.76–13.96)	^***^
Glucose, mmol/L, median (IQR)	5.30 (4.70–6.90)	5.30 (4.70–6.80)	5.30 (4.70–6.70)	0.990
TG, mmol/L, median (IQR)	1.44 (0.94–2.05)	1.27 (0.97–1.84)	1.35 (0.90–2.01)	0.634
TC, mmol/L, median (IQR)	4.57 (3.86–5.28)	4.34 (3.66–5.19)	4.54 (3.79–5.36)	0.412
HDL-C, mmol/L, median (IQR)	1.05 (0.88–1.30)	1.04 (0.86–1.23)	0.98 (0.81–1.20)	0.292
LDL-C, mmol/L, mean ± SD	2.59 ± 0.84	2.56 ± 0.86	2.57 ± 0.90	0.965
ApoA, g/L, median (IQR)	1.28 (1.12–1.45)	1.25 (1.10–1.45)	1.27 (1.07–1.42)	0.447
ApoB, g/L, median (IQR)	0.87(0.71–1.02)	0.84(0.69–1.02)	0.87(0.68–1.03)	0.692
Hcy, μmol/L, median (IQR)	12.98 (10.16–16.45)	13.24 (10.43–16.99)	12.66 (10.29–15.74)	0.719
**Hematoma location, n(%)**				
Frontal lobe	21 (14.38)	24 (16.44)	21 (14.38)	0.875
Parietal lobe	25 (17.12)	13 (8.90)	14 (8.59)	0.055
Temporal lobe	42 (28.77)	49 (33.56)	38 (26.03)	0.360
Occipital lobe	23 (15.75)	22 (15.07)	17 (11.64)	0.559
Basal ganglia	40 (27.40)	50 (34.25)	84 (57.53)	^***^
Brainstem	15 (10.27)	8 (5.48)	8 (5.48)	0.182
Cerebellum	22 (15.07)	14 (9.59)	16 (10.96)	0.322
Concurrent ventricular hemorrhage	12 (8.22)	9 (6.16)	10 (6.85)	0.784
**Neuropsychological function**				
NIHSS1 score, median (IQR)	5 (4–8)	6 (5–10)	7 (5–10)	^***^
NIHSS2 score, median (IQR)	4 (2–6)	5 (2–8)	5 (3–9)	^***^
MMSE score, median (IQR)	22 (17–26)	23 (18–26)	23 (18–26)	0.240
GCS score, median (IQR)	15 (15–15)	15 (15–15)	15 (15–15)	0.479
BI score, median (IQR)	70 (50–90)	65 (50–80)	60 (40–80)	0.001
mRS score, median (IQR)	2 (2–3)	3 (2–4)	3 (2–4)	0.006
HAMD score, median (IQR)	4 (2–6)	5 (2–8)	5 (3–9)	0.024

^***^P < 0.001.

**Table 3:** RDW tertiles of patients.

**Variables**	**Depression (*n* = 93)**	**Non-depression (*n* = 345)**	**χ^2^**	***p* value**
RDW			25.88	^***^
Tertile 1 (10.96–12.95)	11 (11.83%)	135 (39.13%)	24.57	^***^
Tertile 2 (12.97–13.69)	37 (39.78%)	109 (31.59%)	2.21	0.137
Tertile 3 (13.70–16.76)	45 (48.39%)	101 (29.28%)	12.04	0.001

^***^P < 0.001.

### 3.3. Association between the level of RDW and depression after ICH

We conducted the multivariate logistic regression analysis by including WBC, CRP, basal ganglia infarction, RDW, NIHSS1 score, NIHSS2 score, mRS score, and BI score as independent variables, confirming that the NIHSS1 score (OR: 1.404; 95% CI: 1.205–1.635, *p* < 0.001), NIHSS2 score (OR: 1.237; 95% CI: 1.041–1.471, *p* = 0.016), RDW (OR: 2.832; 95% CI: 1.748–4.587; *p* < 0.001) could independently predict depression at 3 months after ICH (Table 4). Correlation analyses indicated the positive relationship between the NIHSS1 scores and the HAMD scores (*r* = 0.283; *p* < 0.001), and the positive relationship between the NIHSS2 scores and the HAMD scores in all patients 3 months after admission (*r* = 0.284, *p* < 0.001). Similarly, a weak positive correlation was found between RDW and CRP (*r* = 0.100, *p*= 0.036). Moreover, a positive correlation also existed between RDW and WBC (*r* = 0.288, *p* < 0.001).

**Table 4:** Multivariate logistic regression analysis for ICH patients' depression.

**Variables**	**OR**	**95% CI**	***p***
WBC	1.124	0.983–1.284	0.087
CRP	1.038	0.842–1.280	0.725
Basal ganglia hematoma	1.377	0.724–2.617	0.329
RDW	2.832	1.748–4.587	^***^
NIHSS1 score	1.404	1.205–1.635	^***^
NIHSS2 score	1.237	1.041–1.471	0.016
BI score	0.839	0.977–1.019	0.251
mRS score	0.998	0.977–1.019	0.839

^***^P < 0.001.

**Figure 2:**
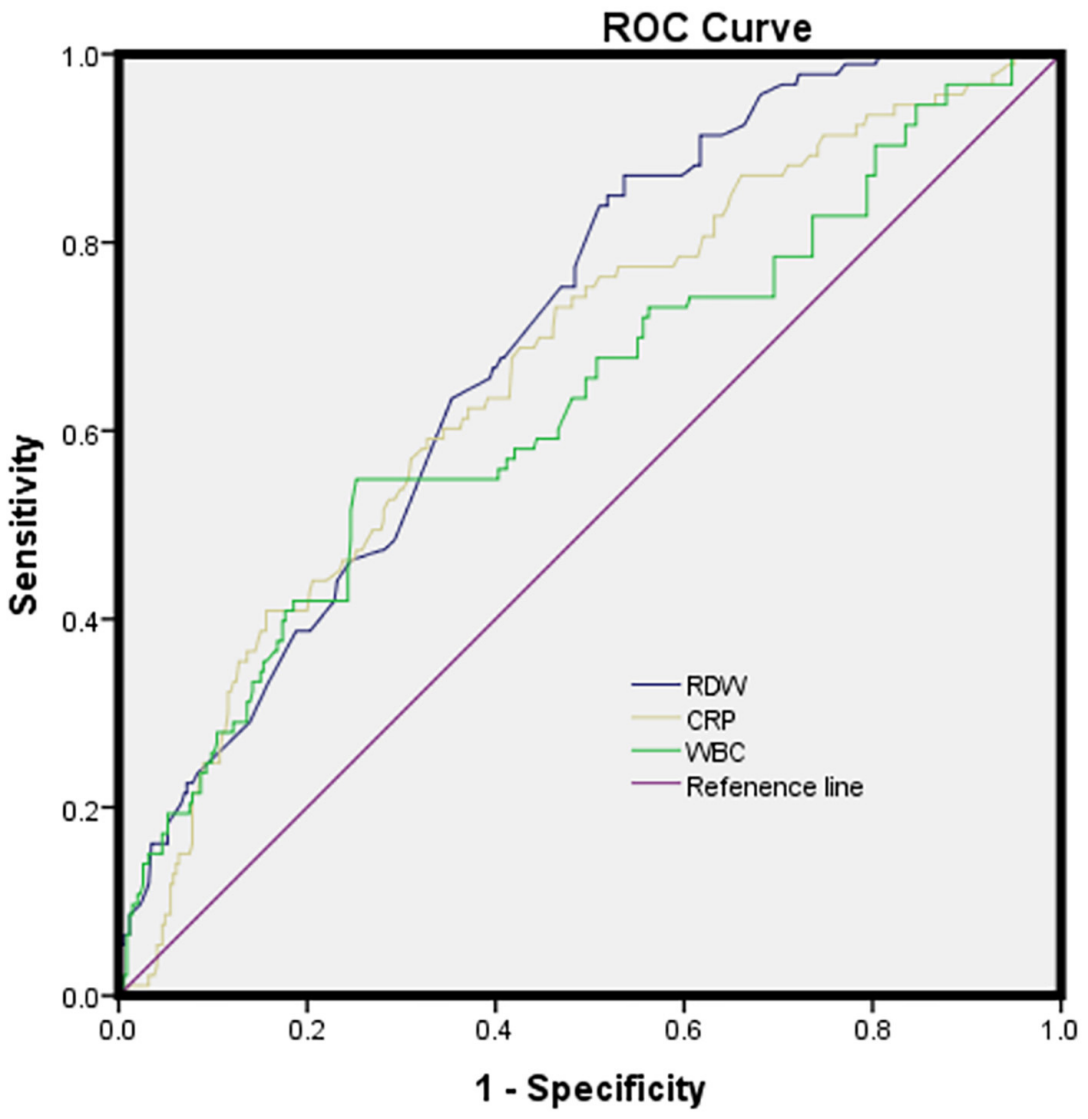
The ROC curves for the prediction of post-ICH depression. Predictive values of RDW, CRP, and WBC for depression at 3 months after ICH: AUC of 0.669 (95%CI, 0.607-0.730; *p* < 0.001) for CRP; 0.631 (95% CI, 0.564–0.698; *p* < 0.001) for WBC; and 0.703 (95% CI, 0.649–0.757; *p* < 0.001) for RDW; RDW had a COV of 13.68, and the sensitivity and specificity were 63.4% and 64.6%, respectively.

In the logistic regression model without making any adjustments and the model with multiple adjustments, we considered all patients as a whole, considering the post-ICH depression and the lowest tertile as the dependent variable and the reference, respectively, for investigating RDW (Table 5). In the logistic regression model without making any adjustments, for patients with admission RDW, the highest tertile presented more obvious ICH depression symptoms relative to the lowest tertile (non-adjusted: OR: 5.468; 95% CI: 2.694–11.098, *p* < 0.001). In the logistic regression model that adjusted for the confounders of age, sex, education years, marital status, vascular risk factors, BGLs, baseline NIHSS score, BI score, mRS score, and laboratory data (WBC and CRP), the highest tertile of RDW could independently predict the prevalence of post-ICH depression (model 1b: OR = 5.336, 95% CI = 2.624–10.852, *p* < 0.001; model 2c: OR = 5.086, 95% CI = 2.482–10.426, *p* < 0.001; model 3d: OR = 4.225, 95% CI = 1.686–10.586, *p* = 0.002). As revealed by the ROC curve, the estimated optimal COV of RDW that predicted the post-ICH depression was 13.68, with the sensitivity and specificity reaching 63.4% and 64.6%, respectively, and the AUC at 0.703 (95% CI: 0.649–0.757; *p* < 0.001) (Figure 2).

**Table 5:** Multivariate logistic regression analysis demonstrating the independent predictors of depression after ICH.

	**Tertile**	**OR^a^**	**95% CI**	***p* value**
Unadjusted	Middle	4.116	2.030–8.549	^***^
	Highest	5.468	2.694–11.098	^***^
Model 1^b^	Middle	4.201	2.039–8.653	^***^
	Highest	5.336	2.624–10.852	^***^
Model 2^c^	Middle	4.195	2.021–8.707	^***^
	Highest	5.086	2.482–10.426	^***^
Model 3^d^	Middle	4.266	1.690–10.770	0.002
	Highest	4.225	1.686–10.586	0.002

^***^P < 0.001.

^a^Reference OR (1.000) is the lowest tertile of RDW for PSD.

^b^Model 1: adjusted for age, sex, education years, and marital status.

^c^Model 2: adjusted for covariates from Model 1, with further adjustment for vascular risk factors.

^d^Model 3: adjusted for covariates from Model 2, with further adjustment for variables with a p < 0.05 in univariate analysis (WBC, CRP, basal ganglia hematoma, baseline NIHSS score, mRS score, and BI score).

## 4. Discussion

To the best of our knowledge, this study is the first prospective cohort study exploring the relationship between RDW and PSD at 3 months in ICH patients. Our results revealed that high-level RDW at admission exhibited an obvious relation to the 3 month-post-ICH depression and documented that, after adjusting for major confounders, patients in the highest RDW tertile presented a post-ICH depression risk that was 4.225-fold higher than those in the lowest RDW tertile, which epidemiologically proved the potential effectiveness of RDW for predicting the post-ICH depression at 3 months. Hence, RDW can serve well as a useful therapeutic target for treating depression after ICH.

A prospective nationwide hospital-based cohort study revealed that the incidence of adverse events in ICH patients was 2.5 times higher than that of IS patients (52.0 % vs. 21.7 %, respectively) (33). In fact, 21.23% of ICH patients in this study developed depression in the third month, meeting the results of previous studies (34). According to previous investigations, the frequency of PSD was in the range of 21.2%−75% (35, 36). The possible reasons for the difference in the incidence of PSD are related to the different criteria for determining PSD, the methods of psychological assessment, and the time of assessment. PSD can be diagnosed dynamically, and different time points impact the prevalence of PSD in different ways. The study revealed the relationship between high-level RDW and higher NIHSS1 and NIHSS2 scores in ICH patients. As found in the study, relative to patients in the non-depression group, for ICH patients in the depression group, the stroke severity was elevated and functional outcomes were poor, which matched the previous studies (37). The frontal lobe, the temporal lobe, and the basal ganglia act as the core brain regions in the emotional network, and the appearance of hemorrhage in these brain regions is associated with PSD. Damage to the limbic–cortical–striatal–pallidal–thalamic (LCSPT) circuit can lead to depressive symptoms (38, 39). We also found similar results that patients who had post-ICH depression held a larger likelihood of developing basal ganglia hematoma. Nevertheless, patients in the depression and non-depression groups after ICH did not present obvious differences in the frontal and temporal lobe lesions. A larger-sized sample needs to be adopted for validating the abovementioned findings.

Research has not explored well the mechanism by which the elevated RDW level was accompanied by an increased incidence rate of depression. Hence, discussing the possible physiological or pathological effects of elevated RDW levels on depression development is of larger significance. At first, numerous studies have shown that RDW may be associated with the inflammatory response after ICH (18, 40). The cascade of inflammatory reactions caused by direct mechanical compression of the surrounding tissues by the hematoma, chemical reactions, and oxidative damage after ICH is the cause of secondary neuronal death around the hematoma (41). Animal experiments and clinical studies have demonstrated that hematoma causes an early inflammatory response that exacerbates brain tissue damage, thereby affecting patients' prognosis (41, 42). Inflammatory cytokines such as TNF-α, IL-1β, and IL-6 inhibit erythropoietin-induced erythrocyte maturation, resulting in the entry of immature erythrocytes into the peripheral blood, which can lead to increased RDW levels (43). Shafiee et al. demonstrated that elevated RDW is associated with WBC, strongly supporting the consideration of RDW as an effective marker of inflammation (44). Some studies have suggested that RDW is similar to TNF receptors or CRP, which are also markers for inflammation (45). Ferrucci et al. proposed that various inflammatory cytokines can be used as a parameter, demonstrating that higher levels of inflammation and high RDW levels in non-anemic elderly are strongly associated with erythropoietin production but are negatively related to anemia (46). Additionally, pro-inflammatory cytokines reduce erythrocyte survival by altering the deformability of the erythrocyte membrane and inhibiting erythropoiesis, ultimately leading to erythrocyte injury. The higher RDW reports erythropoiesis impairment, manifested by chronic inflammation and elevated oxidative stress (47). Besides, previous studies have demonstrated the impact of RDW on ICH occurrence and prognosis, revealing its vital effect on the unfavorable prognostic outcome after ICH (48). Nevertheless, depressive symptoms report weak functional outcomes for ICH patients (49).

A second explanation is that oxidative stress has been considered a significant factor determining RDW, which is capable of lowering erythrocytes' lifespan, while inflammation shows a close association with inhibited erythropoiesis. Both oxidative stress and inflammation can elevate the RDW level (50–52). Oxidative stress may considerably affect the course of ICH and lead to elevated RDW. Low antioxidant defenses present a positive relation to RDW and can also independently predict all-cause mortality, especially in ICH (53). In addition, after cerebral vessels are broken, blood components, composed of erythrocytes and their metabolites, thrombin, and fibrinogen, are capable of entering the cerebral parenchyma through the impaired blood–brain barrier (BBB), as well as inducing inflammatory cascades and oxidative stress. These exacerbate the destruction of the BBB and promote blood components to be further infiltrated, thereby causing a vicious cycle exacerbating the neurological damage as well as the brain edema caused by ICH (54). Similarly, elevated RDW inhibits endothelium-dependent nitric oxide-mediated vasodilation, reducing the oxygen supply to the impaired brain tissue and thus diminishing the repairing and recovery ability of the nervous system (55).

Besides, the inflammatory response remarkably affects ICH. Upon the occurrence of ICH, the organism is capable of inducing a systemic inflammatory response while elevating the RDW, WBC, and CRP levels in ICH patients (40, 56, 57). Furthermore, previous reports demonstrated that high RDW reported increased inflammation indices, such as CRP and IL-6 (58, 59). A meta-analysis revealed that patients with higher CRP levels in the acute phase of stroke are more likely to develop PSD (13). According to Gong et al., the higher the WBC level, the more obvious the depressive symptoms after ICH (34). As found in the study, RDW could robustly predict post-ICH depression (AUC: 0.665) compared to CRP (AUC: 0.697) and WBC (AUC: 0.692). In addition, inflammation mediates the neurotransmitter alteration, especially the synthesis and the metabolism of glutamine and 5-hydroxytryptamine, thereby contributing to the dysfunction of synaptic plasticity and depression. RDW is capable of reflecting the inflammatory state after ICH, on the one hand, and can affect depression development, on the other hand (60). Based on the AUC of RDW, RDW can well predict the prognosis of depression after ICH, and the sensitivity and specificity are 63.4% and 64.6%, respectively. Taken together, elevated RDW levels may report high inflammation levels, which exhibits a larger possibility of leading to depression after ICH. Consequently, patients with elevated RDW levels should be treated with caution. Further prospective studies are vital to investigating whether RDW lowering treatment offers a potential prevention or therapeutic target for depression after ICH. We believe that the use of RDW could be standardized when estimating depression in patients with ICH, as RDW is a laboratory parameter automatically provided in conventional hemograms.

The study has some limitations: (1) The measurement of the RDW level only occurred at the acute stage of ICH patients; hence, the study failed to obtain the data on the time and duration for the biomarkers to make a change in these participants, (2) the study only assessed the post-ICH depression once, at the 3-month follow-up. Hence, the study will be more valid if the assessment can be conducted both at shorter (1 month and 2 months) and longer time (1 year and 2 years) points, (3) the study excluded patients who had serious dysarthria or aphasia; as a result, the actual prevalence of post-ICH depression may be underestimated and result bias may occur, (4) we did not investigate the availability of antidepressant treatment during the follow-up period from onset to 3 months, thereby influencing the incidence of post-ICH depression, and (5) several studies have already reported the association of several risk factors with post-ICH depression, such as economic status and social support.

## 5. Conclusions

In summary, a higher RDW can independently report post-ICH depression at 3 months, showing that RDW on admission may be an important biomarker of inflammatory-related disease for the prediction of post-ICH depression occurrence.

## Data availability statement

The raw data supporting the conclusions of this article will be made available by the authors, without undue reservation.

## Ethics statement

The studies involving human participants were reviewed and approved by Bozhou Hospital Affiliated to Anhui Medical University. Participants provided informed consent prior to inclusion in this study.

## Author contributions

XZ and YL designed the research study and performed the research. LM, YL, and XZ analyzed the data. YL wrote the manuscript. All authors contributed to editorial changes in the manuscript and read and approved the final manuscript.

## References

[1] Pasi M Sugita L Xiong L Charidimou A Boulouis G Pongpitakmetha T. Association of cerebral small vessel disease and cognitive decline after intracerebral hemorrhage. Neurology. (2021) 96:e182–92. 10.1212/WNL.0000000000011050 33067403 PMC7905779

[2] Tu WJ Hua Y Yan F Bian H Yang Y Lou M. Prevalence of stroke in China, 2013-2019: a population-based study. Lancet Reg Health West Pac. (2022) 28:100550. 10.1016/j.lanwpc.2022.100550 36507089 PMC9727498

[3] Lopatkiewicz AM Pera J Slowik A Dziedzic T. Association of early and later depressive symptoms with functional outcome after ischemic stroke. J Neural Transw (Vienna). (2021) 128:679–86. 10.1007/s00702-021-02328-w 33728483 PMC8105243

[4] Blöchl Maria Meissner Sophie Nestler Steffen. Does depression after stroke negatively influence physical disability? a systematic review and meta-analysis of longitudinal studies. J Affect Disord. (2019) 247:45–56. 10.1016/j.jad.2018.12.082 30654265

[5] Kang C. Predictors of post-stroke cognition among geriatric patients: the role of demographics, pre-stroke cognition, and trajectories of depression. Front Psychol. (2021) 12:717817. 10.3389/fpsyg.2021.717817 34381407 PMC8349975

[6] Koivunen RJ Harno H Tatlisumak T Putaala J. Depression, anxiety, and cognitive functioning after intracerebral hemorrhage. Acta Neurol Scand. (2015) 132:179–84. 10.1111/ane.12367 25639837

[7] Stern-Nezer S Eyngorn I Mlynash M Snider RW Venkatsubramanian C Wijman CAC. Depression one year after hemorrhagic stroke is associated with late worsening of outcomes. NeuroRehabilitation. (2017) 41:179–87. 10.3233/NRE-171470 28505996

[8] Cheng Y Wang Y Wang X Jiang Z Zhu L Fang S. Neutrophil-to-lymphocyte ratio, platelet-to-lymphocyte ratio, and monocyte-to-lymphocyte ratio in depression: an updated systematic review and meta-analysis. Front Psychiatry. (2022) 13:893097. 10.3389/fpsyt.2022.893097 35782448 PMC9240476

[9] Moylan S Maes M Wray NR Berk M. The neuroprogressive nature of major depressive disorder: pathways to disease evolution and resistance, and therapeutic implications. Mol Psychiatry. (2013) 18:595–606. 10.1038/mp.2012.33 22525486

[10] Leonard B Maes M. Mechanistic explanations how cell-mediated immune activation, inflammation and oxidative and nitrosative stress pathways and their sequels and concomitants play a role in the pathophysiology of unipolar depression. NeurosciBiobehav Rev. (2012) 36:764–85. 10.1016/j.neubiorev.2011.12.005 22197082

[11] Su JA Chou SY Tsai CS Hung TH. Cytokine changes in the pathophysiology of post stroke depression. Gen Hosp Psychiatry. (2012) 34:35–9. 10.1016/j.genhosppsych.2011.09.020 22055333

[12] Kang HJ Bae KY Kim SW Kim JT Park MS Cho KH. Effects of interleukin-6, interleukin-18, and statin use, evaluated at acute stroke, on post-stroke depression during 1-year follow up. Psychoneuroendocrinology. (2016) 72:156–60. 10.1016/j.psyneuen.2016.07.001 27428088

[13] Yang Y Zhu L Zhang B Gao J Zhao T Fang S. Higher levels of C-reactive protein in the acute phase of stroke indicate an increased risk for post-stroke depression: a systematic review and meta-analysis. Neurosci Biobehav Rev. (2022) 134:104309. 10.1016/j.neubiorev.2021.08.018 34416242

[14] Gibney SM McGuinness B Prendergast C Harkin A Connor TJ. Poly I:C-induced activation of the immune response is accompanied by depression and anxiety-like behaviours, kynurenine pathway activation and reduced BDNF expression. Brain Behav Immun. (2013) 28:170–81. 10.1016/j.bbi.2012.11.010 23201589

[15] Hu J Wang L Fan K Ren W Wang Q Ruan Y. The association between systemic inflammatory markers and post-stroke depression: a prospective stroke cohort. Clin Interv Aging. (2021) 16:1231–9. 10.2147/CIA.S314131 34234423 PMC8243596

[16] Wu Y Wang L Hu K Yu C Zhu Y Zhang S. Mechanisms and therapeutic targets of depression after intracerebral hemorrhage. Front Psychiatry. (2018) 9:682. 10.3389/fpsyt.2018.00682 30618863 PMC6304443

[17] Salvagno GL Sanchis-Gomar F Picanza A Lippi G. Red blood cell distribution width: a simple parameter with multiple clinical applications. Crit Rev Clin Lab Sci. (2015) 52:86–105. 10.3109/10408363.2014.992064 25535770

[18] Cui Z Liu C Sun G Huang L Zhou W A. prognostic nomogram incorporating red cell distribution width for patients with intracerebral hemorrhage. Medicine (Baltimore). (2020) 99:e23557. 10.1097/MD.0000000000023557 33327308 PMC7738053

[19] Abrahan LL Ramos JDA Cunanan EL Tiongson MDA Punzalan FER. Red cell distribution width and mortality in patients with acute coronary syndrome: a meta-analysis on prognosis. Cardiol Res. (2018) 9:144–52. 10.14740/cr732w 29904449 PMC5997444

[20] Felker GM Allen LA Pocock SJ Shaw LK McMurray JJ Pfeffer MA. Red cell distribution width as a novel prognostic marker in heart failure: data from the CHARM Program and the Duke Databank. J Am Coll Cardiol. (2007) 50:40–7. 10.1016/j.jacc.2007.02.067 17601544

[21] Pinho J Marques SA Freitas E Araújo J Taveira M Alves JN. Red cell distribution width as a predictor of 1-year survival in ischemic stroke patients treated with intravenous thrombolysis. Thromb Res. (2018) 164:4–8. 10.1016/j.thromres.2018.02.002 29438871

[22] Chugh C Nyirjesy SC Nawalinski KP Sandsmark DK Frangos S Maloney-Wilensky E. Red blood cell distribution width is associated with poor clinical outcome after subarachnoid hemorrhage: a pilot study. Neurocrit Care. (2015) 23:217–24. 10.1007/s12028-015-0117-x 25672971

[23] Li Y Zhang M Dong C Xue M Li J Wu G. Elevated red blood cell distribution width levels at admission predicts depression after acute ischemic stroke: a 3-month follow-up study. Neuropsychiatr Dis Treat. (2022) 18:695–704. 10.2147/NDT.S351136 35391945 PMC8979940

[24] Dai M Wei Q Zhang Y Fang C Qu P Cao L. Predictive value of red blood cell distribution width in poststroke depression. Comput Math Methods Med. (2021) 83, 61504. 10.1155/2021/8361504 34335867 PMC8315889

[25] Demircan F Gözel N Kilinç F Ulu R Atmaca M. The Impact of Red Blood Cell Distribution Width and Neutrophil/Lymphocyte Ratio on the Diagnosis of Major Depressive Disorder. Neurol Ther. (2016) 5:27–33. 10.1007/s40120-015-0039-8 26686339 PMC4919129

[26] Ani C Ovbiagele B. Elevated red blood cell distribution width predicts mortality in persons with known stroke. J Neurol Sci. (2009) 277:103–8. 10.1016/j.jns.2008.10.024 19028393

[27] Feng GH Li HP Li QL Fu Y Huang RB. Red blood cell distribution width and ischaemic stroke. Stroke Vasc Neurol. (2017) 2:172–5. 10.1136/svn-2017-000071 28989807 PMC5628378

[28] Song SY Hua C Dornbors D Kang RJ Zhao XX Du X. Baseline red blood cell distribution width as a predictor of stroke occurrence and outcome: a comprehensive meta-analysis of 31 studies. Front Neurol. (2019) 10:1237. 10.3389/fneur.2019.01237 31849813 PMC6901990

[29] Bonita R Beaglehole R. Modification of Rankin scale: recovery of motor function after stroke. Stroke. (1989) 19:1497–500. 10.1161/01.STR.19.12.1497 3201508

[30] Mahorney F. Functional evaluation: the Barthel index. Md State Med J. (1965) 14:61–5. 14258950

[31] Ridha B Rossor M. The mini mental state examination. Pract Neurol. (2010) 5:298–303. 10.1111/j.1474-7766.2005.00333.x

[32] Hamilton M A. rating scale for depression. J Neurol Neurosurg Psychiatry. (1960) 23:56–62. 14399272 10.1136/jnnp.23.1.56 PMC495331

[33] Tu WJ Chao BH Ma L Yan F Cao L Qiu H. Case-fatality, disability and recurrence rates after first-ever stroke: a study from bigdata observatory platform for stroke of China. Brain Res Bull. (2021) 175:130–5. 10.1016/j.brainresbull.2021.07.020 34329730

[34] Gong X Lu Z Feng X Yu C Xue M Yu L. Elevated Neutrophil-to-Lymphocyte Ratio Predicts Depression After Intracerebral Hemorrhage. Neuropsychiatr Dis Treat. (2020) 16:2153–9. 10.2147/NDT.S269210 33061386 PMC7518785

[35] Huang G Chen H Wang Q Hong X Hu P Xiao M. High platelet-to-lymphocyte ratio are associated with post-stroke depression. J Affect Disord. (2019) 246:105–11. 10.1016/j.jad.2018.12.012 30578944

[36] Khazaal W Taliani M Boutros C Abou-Abbas L Hosseini H Salameh P. Psychological complications at 3 months following stroke: prevalence and correlates among stroke survivors in Lebanon. Front Psychol. (2021) 12:663267. 10.3389/fpsyg.2021.663267 34177717 PMC8222528

[37] Ayerbe L Ayis S Wolfe CD Rudd AG. Natural history, predictors and outcomes of depression after stroke: systematic review and meta-analysis. Br J Psychiatry. (2013) 202:14–21. 10.1192/bjp.bp.111.107664 23284148

[38] Terroni L Amaro E Iosifescu DV Tinone G Sato JR Leite CC. Stroke lesion in cortical neural circuits and post-stroke incidence of major depressive episode: a 4-month prospective study World. J Biol Psychiatry. (2011) 12:539–48. 10.3109/15622975.2011.562242 21486107 PMC3279135

[39] Jastorff J Huang YA Giese MA Vandenbulcke M. Common neural correlates of emotion perception in humans. Hum Brain Mapp. (2015) 36:4184–201. 10.1002/hbm.22910 26219630 PMC6869080

[40] Lorente L Martín MM González-Rivero AF Pérez-Cejas A Sabatel R Ramos L. Red blood cell distribution width and mortality of spontaneous intracerebral hemorrhage patients. Clin Neurol Neurosurg. (2020) 195:106066. 10.1016/j.clineuro.2020.106066 32652396

[41] Zhou Y Wang Y Wang J Anne Stetler R Yang QW. Inflammation in intracerebral hemorrhage: from mechanisms to clinical translation. Prog Neurobiol. (2014) 115:25–44. 10.1016/j.pneurobio.2013.11.003 24291544

[42] Mracsko E Veltkamp R. Neuroinflammation after intracerebral hemorrhage. Front Cell Neurosci. (2014) 8:388. 10.3389/fncel.2014.00388 25477782 PMC4238323

[43] Kalyani P Jamil K A. study on biochemical facet of anemia in cancers: A strong link between erythropoietin and tumor necrosis factor alpha in anemic cancer patients. Indian J Cancer. (2015) 52:127–32. 10.4103/0019-509X.175579 26838000

[44] Shafiee M Tayefi M Hassanian SM Ghaneifar Z Parizadeh MR Avan A. Depression and anxiety symptoms are associated with white blood cell count and red cell distribution width: A sex-stratified analysis in a population-based study. Psychoneuroendocrinology. (2017) 84:101–8. 10.1016/j.psyneuen.2017.06.021 28697416

[45] Macdougall IC Cooper A. The inflammatory response and epoetin sensitivity. Nephrol Dial Transplant. (2002) 17:48–52. 10.1093/ndt/17.suppl_1.48 11812913

[46] Ferrucci L Guralnik JM Woodman RC Bandinelli S Lauretani F Corsi AM. Proinflammatory state and circulating erythropoietin in persons with and without anemia. Am J Med. (2005) 118:1288. 10.1016/j.amjmed.2005.06.039 16271918

[47] Dada OA Uche E Akinbami A Odesanya M John-Olabode S Adediran A. The relationship between red blood cell distribution width and blood pressure in patients with type 2 diabetes mellitus in Lagos, Nigeria. J Blood Med. (2014) 5:185–9. 10.2147/JBM.S67989 25278786 PMC4179754

[48] He M Wang H Tang Y Cui B Xu B Niu X. Red blood cell distribution width in different time-points of peripheral thrombolysis period in acute ischemic stroke is associated with prognosis. Aging (Albany NY). (2022) 14:5749–67. 10.18632/aging.204174 35832033 PMC9365566

[49] Sallinen H Sairanen T Strbian D. Quality of life and depression 3 months after intracerebral hemorrhage. Brain Behav. (2019) 9:e01270. 10.1002/brb3.1270 30907075 PMC6520301

[50] Montagnana M Cervellin G Meschi T Lippi G. The role of red blood cell distribution width in cardiovascular and thrombotic disorders. Clin Chem Lab Med. (2011) 50:635–41. 10.1515/cclm.2011.831 22505527

[51] Marinkovic D Zhang X Yalcin S Luciano JP Brugnara C Huber T. Foxo3 is required for the regulation of oxidative stress in erythropoiesis. J Clin Invest. (2007) 117:2133–44. 10.1172/JCI31807 17671650 PMC1934587

[52] Li N Zhou H Tang Q. Red Blood Cell Distribution Width: A Novel Predictive Indicator for Cardiovascular and Cerebrovascular Diseases. Dis Markers. (2017) 2017:7089493. 10.1155/2017/7089493 29038615 PMC5606102

[53] Friedman JS Lopez MF Fleming MD Rivera A Martin FM Welsh ML. SOD2-deficiency anemia: protein oxidation and altered protein expression reveal targets of damage, stress response, and antioxidant responsiveness. Blood. (2004) 104:2565–73. 10.1182/blood-2003-11-3858 15205258

[54] Chen S Li L Peng C Bian C Ocak PE Zhang JH. Targeting Oxidative Stress and Inflammatory Response for Blood-Brain Barrier Protection in Intracerebral Hemorrhage. Antioxid Redox Signal. (2022) 37:115–34. 10.1089/ars.2021.0072 35383484

[55] Kim-Shapiro DB Schechter AN Gladwin MT. Unraveling the reactions of nitric oxide, nitrite, and hemoglobin in physiology and therapeutics. Arterioscler Thromb Vasc Biol. (2006) 26: 697– 705. 10.1161/01.ATV.0000204350.44226.9a 16424350

[56] Wang D Wang J Li Z Gu H Yang K Zhao X. C-reaction protein and the severity of intracerebral hemorrhage: a study from chinese stroke center alliance. Neurol Res. (2022) 44:285–90. 10.1080/01616412.2021.1980842 34559025

[57] Leasure AC Kuohn LR Vanent KN Bevers MB Kimberly WT Steiner T. association of serum IL-6 (Interleukin 6) with functional outcome after intracerebral hemorrhage. Stroke. (2021) 52:1733–40. 10.1161/STROKEAHA.120.032888 33682454 PMC8085132

[58] Li G Jia P Zhao J Wu X Duan Y Liu D. Usefulness of RBC distribution width and C-reactive protein to predict mortality in pediatric non-cardiac critical illness. Am J Emerg Med. (2019) 37:2143–50. 10.1016/j.ajem.2019.01.041 30772131

[59] Miyamoto K Inai K Takeuchi D Shinohara T Nakanishi T. Relationships among red cell distribution width, anemia, and interleukin-6 in adult congenital heart disease. Circ J. (2015) 79:1100–6. 10.1253/circj.CJ-14-1296 25740502

[60] Müller N Schwarz MJ. The immune-mediated alteration of serotonin and glutamate: towards an integrated view of depression. Mol Psychiatry. (2007) 12:988–1000. 10.1038/sj.mp.4002006 17457312

